# Hand dominance and experience improve bimanual performance on arthroscopic simulator task

**DOI:** 10.1007/s00167-022-06920-9

**Published:** 2022-02-25

**Authors:** Aoife A. Feeley, John P. Gibbons, Iain H. Feeley, Eilis Fitzgerald, Khalid Merghani, Eoin Sheehan

**Affiliations:** 1 Department of Orthopaedics, Midlands Regional Hospital Tullamore, Arden Road, Tullamore, Co. Offaly Ireland; 2 Royal College of Surgeons Ireland, Dublin, Ireland; 3 Department of Orthopaedics, Tallaght University Hospital, Dublin, Ireland; 4 Department of Orthopaedics, National Orthopaedic Hospital Cappagh, Dublin, Ireland

**Keywords:** Simulation, Surgical training, Handedness, Bi-manual control

## Abstract

**Purpose:**

The aim of this study was to identify if experience in arthroscopy confers ambidexterity to the operator and the role of baseline characteristics in arthroscopic simulator performance.

**Methods:**

A prospective comparative study was carried out across four regional Orthopaedic training centres. Participants were divided into novice, intermediate or experienced groups based on arthroscopic experience. Baseline demographics including age, sex, handedness, and gaming history were also collected. Following familiarisation with the procedure, participants were asked to complete a simulated task requiring bimanual control consisting of visualisation with camera control and manipulation of highlighted objects using a grasping instrument. One attempt using camera control and grasping accuracy per hand was performed by each participant, with scores for each hand collected for analysis. Performance scores for camera alignment, camera path length, grasper path length and grasping efficiency were collected. Time taken to completion was also noted for each attempt.

**Results:**

Fifty-six participants were recruited to the study. A significant difference in grasping efficiency between groups in the dominant hand was demonstrated (*p* = 0.013). Novices demonstrated laterality with superior performance in grasping efficiency in the dominant hand (*p* = 0.001). No significant difference was noted between dominant and non-dominant hand performance in the experienced group.

**Conclusion:**

Arthroscopic simulation-based training is a valuable learning tool for orthopaedic training. This study demonstrated that experienced orthopaedic surgeons have a greater degree of ambidexterity than intermediate or novice groups, hypothesised by authors to be conferred through conventional orthopaedic training. Dedicated bimanual control tasks to reduce laterality in trainees should be incorporated in simulated surgical curricula.

**Level of evidence:**

III.

## Introduction

Surgery is a bimanual profession; it involves adequate visualisation, manipulation, and mobilisation of structures held within enclosed spaces. Execution of procedural tasks efficiently is the hallmark of a fully qualified surgeon. Research to date has focused on the acquisition of dexterity in the non-dominant hand of general surgeons, a skillset accentuated through the introduction of minimally invasive surgery into standard practice. The bimanual control required for visualisation and manipulation of structures in the abdomen has been the subject of much of the research in this area, with evidence on the superiority of left handed-people noted in dexterous tasks, often theorised to arise from an adaptation to a right-hand dominant environment [4]. Efforts to mitigate the potential impact of hand dominance on surgical performance have resulted in the introduction of robotic assistance to help surgeons overcome limitations of the non-dominant hand [7].

Orthopaedic surgery is lateralised by the nature of the operating field, including the differences in surgical approaches based on the operative side during limb and spinal surgeries, and surgeon handedness. Handedness has been demonstrated to impact patient outcomes in a variety of subspecialties; with hand dominance impacting the quality of pedicle screw placement [19], medialisation of hip implantations [8, 12], and patient outcomes following total knee arthroplasties [9]. However, the degree of ambidexterity accrued through orthopaedic surgical training with both open and arthroscopic exposure has yet to be investigated.

Development of incorporated surgical simulation-based training is an area of burgeoning interest in orthopaedics, with minimally invasive surgical skill acquisition reported to transfer well from the virtual platform in a multitude of specialties including general surgery [18] and gynaecology [17]. Performance demonstrated in expert and novice surgeons in simulated arthroscopic procedures has been evaluated to identify transfer validity of simulation-based modules into orthopaedic practice [10]. While waypoints to evaluate the acquisition of arthroscopic technical skills through simulation training are varied [10]; the use of virtual reality in conferring skills required for arthroscopic surgery has been demonstrated to be an effective training method to prepare trainees for future arthroscopic procedures [5].

The aim of this study was to identify if experience in arthroscopy confers ambidexterity to the operator and if baseline characteristics impact arthroscopic simulator performance. Authors hypothesise that experienced orthopaedic surgeons in the use of arthroscopy will demonstrate enhanced bimanual control compared to those without arthroscopic experience, and that left-hand dominant participants would demonstrate a higher level of bimanuality, indicating that future iterations of simulation-based Orthopaedic training should include dedicated bimanual training to accelerate technical skills in Orthopaedic residents.

## Materials and methods

### Study population

Local institutional board approval was obtained from Midland Regional Hospital Tullamore. Information regarding the study was disseminated to four regional orthopaedic training centres, with doctors undergoing rotation at each invited to participate in this study. Application of inclusion and exclusion criteria were applied (Table 1), 56 participants eligible to participate were stratified into groups according to self-reported experience in arthroscopy. Individuals with no previous arthroscopic experience were categorised as a novice (N), with the intermediate group (I) containing participants reporting between 10 and 100 arthroscopic procedures as primary operator. The experienced group (E) consisted of Orthopaedic surgeons with $\ge$ 100 arthroscopic procedures completed as primary operator. Baseline demographics were collected including age, gender, and handedness. Any previous experience in laparoscopic procedures was also collected for analysis.

**Table 1:** Inclusion and Exclusion criteria

Inclusion criteria	Exclusion criteria
Subjects able and willing to give consent and to comply with the requirements of the study protocol	Volunteers unable to perform the simulated task with both dominant and non-dominant hands
Medical students undergoing clinical rotations in Surgery available for the study time period	Volunteers with motion sickness such that they would be unable to navigate the instruments on screen
Doctors in Orthopaedic posts available on-site	

### Simulator task

Following orientation on the VirtaMed arthroscopic simulator (Zurich, Switzerland) participants in all groups were asked to complete a task on the Fundamental Arthroscopic Surgical Training (FAST) set. The arthroscopic simulator was formulated to deconstruct arthroscopic skill such that each can be learned individually for a gradual accrual of the surgical skillset required to perform an arthroscopy safely. Ergonomics of the basic dome are formulated to mimic conventional portal distance. The simulator has the provision of 30- and 70-degree arthroscope for both knee and hip training modules.

The task chosen for the study mimics the removal of loose bodies from a joint space, with 10 virtual stars placed inside a highlighted field within the basic dome parameters, with visual feedback provided via a screen placed directly above the dome. The task requires bimanual control with camera operation and the use of grasper to visualise, identify, and remove the 10 stars from the highlighted space and release them outside of the dome capsule. To ensure all required steps are completed, the module is programmed to recognise the star as “removed” from the basic dome once the grasper handle has been released outside the confines of the dome.

Participants performed the task once with independent reviewer guidance to familiarise themselves with the equipment and steps required to complete the task. Randomisation was used to determine the hand participants would use for the familiarisation task. Following the familiarisation task attempt, participants were asked to complete the module a further two times, once with the grasper in their dominant (D) hand with camera control operated with their non-dominant (ND) hand, and one attempt completed with the grasper in their non-dominant hand with camera operation under dominant hand control. Participants were asked to complete the tasks in succession, with minimal disruption between attempts to reduce biases.

Simulator software metrics from each attempt were collected following completion of each simulated task including exercise duration (s); camera alignment (%); camera path length (cm); grasper path length (cm); grasper efficiency (%); and distance travelled with grasper jaws open (cm). Measurements were uploaded to SPSS with scores recorded to one decimal place for distance, and two decimals for time. Percentages were recorded as whole numbers for the purposes of this study.

### Statistical analysis

Statistical analysis was carried out using the IBM SPSS software (IBM Inc.). Sample size calculations using effect sizes in previous research on differences in performance across experience was used with an alpha error probability of 0.05 and powering of 0.8 was used. Sample size calculated was 36, with an attrition rate of 10% indicating a minimum of 40 participants were required across groups. Descriptive statistics were expressed as mean ± SD. Reliability testing by computing Cronbach’s Alpha was carried out. Inter-rater reliability was assessed via the intraclass correlation coefficient (ICC) calculated. The one-way ANOVA was used to find the significance among the three groups. For the analysis of baseline dominant hand score, MannWhitney *U* testing was used to assess D and ND control in each dependent factor identified in each group with the exception of time, which was evaluated using independent *t* test with normal distribution identified using Shapiro Wilk’s test for normality. Multivariate tests with Post Hoc tests Bonferroni and Tukey HSD were applied where available. A difference in intra-subject and inter-subject findings was considered statistically significant if *p* < 0.05.

## Results

Fifty-six participants were recruited to this study, with five participants unable to complete the tasks and excluded from analysis. The novice group consisted primarily of medical students undergoing clinical rotations in surgery and interns, while intermediate and experienced groups consisted primarily of residents and fully trained Orthopaedic consultants. Baseline characteristics were demonstrated in Table 2.

**Table 2:** Group characteristics

Group	Age (mean)	Gender (M)	Handedness (RHD)	Gaming history	Arthroscopic experience
Novice (*n* = 20)	25 $\pm$ 4	12/20	16/20	7/20	0
Intermediate (*n* = 22)	32 $\pm 7$7	18/22	20/22	4/22	14.4
Experienced (*n* = 9)	50 $\pm$ 12	9/9	8/9	2/9	> 120

### Task performance

#### Within-subject performance

Participants in the novice group demonstrated a significant difference in grasp efficiency demonstrated in their D and ND attempts (57% $\pm$ 24% vs. 44% $\pm$ 21%, *p* = 0.001), with the dominant hand of each participant significantly outperforming the non-dominant attempt.

**Fig. 1:**
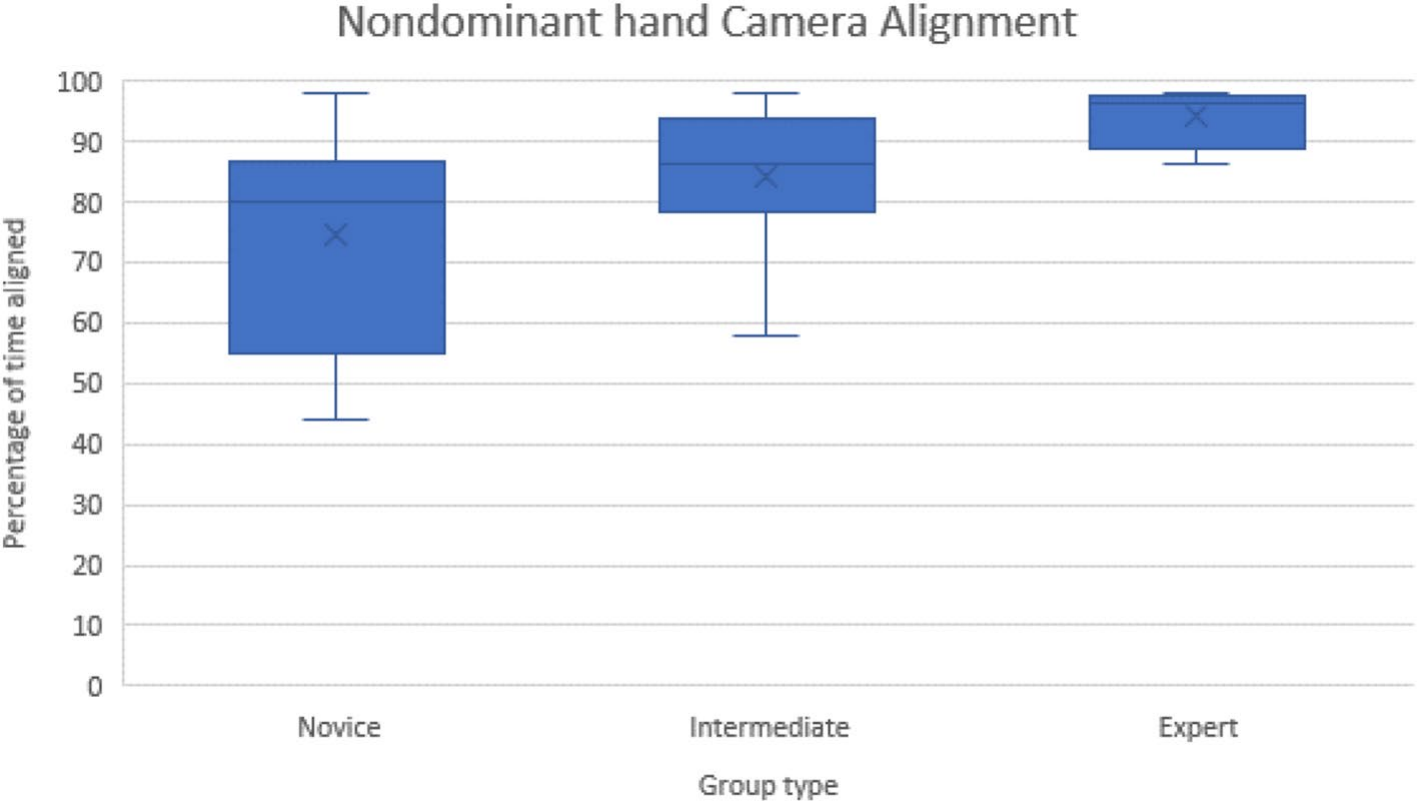
Non-dominant camera alignment between groups. Experts demonstrated consistently better camera alignment with a narrower standard deviation than both novice and intermediate groups

Camera path length, grasper length, grasping efficiency and camera alignment performances were not lateralised in participants in either the intermediate or experienced groups. The experienced group demonstrated a greater degree of within-group precision (Fig. 1).

#### Between-group performance

One-way ANOVA evaluating between-subjects effects indicated grasping efficiency in the dominant hand demonstrated a significant difference (N 44% $\pm 17$ vs. I 55% $\pm$ 23 vs. E 55% $\pm$ 14, *p* = 0.013). Between-group analysis noted a significant difference in length of the camera path during the ND task attempt (N 122.3 cm $\pm$ 162.4 cm vs. I 79.2 cm $\pm$ 32.4 cm vs. E 83.3 cm $\pm$ 35.9 cm, *p* = 0.006) between groups.

### Participant characteristics

#### Hand dominance

Significant difference between left (LHD) and right (RHD) hand dominant participants was noted in two parameters. Length of grasper path was noted to be significantly longer in the LHD dominant group (N 409.1 cm $\pm$ 232.6 cm vs. I 325.1 cm $\pm$ 81.6 cm, *p* = 0.032). Similarly, grasper path length travelled with grasper open was also found to be significantly longer in this participant cohort (N 248.2 cm $\pm$ 167.8 cm vs. I 197.7 cm $\pm$ 93.3 cm, *p* = 0.034).

#### Gender

**Fig. 2:**
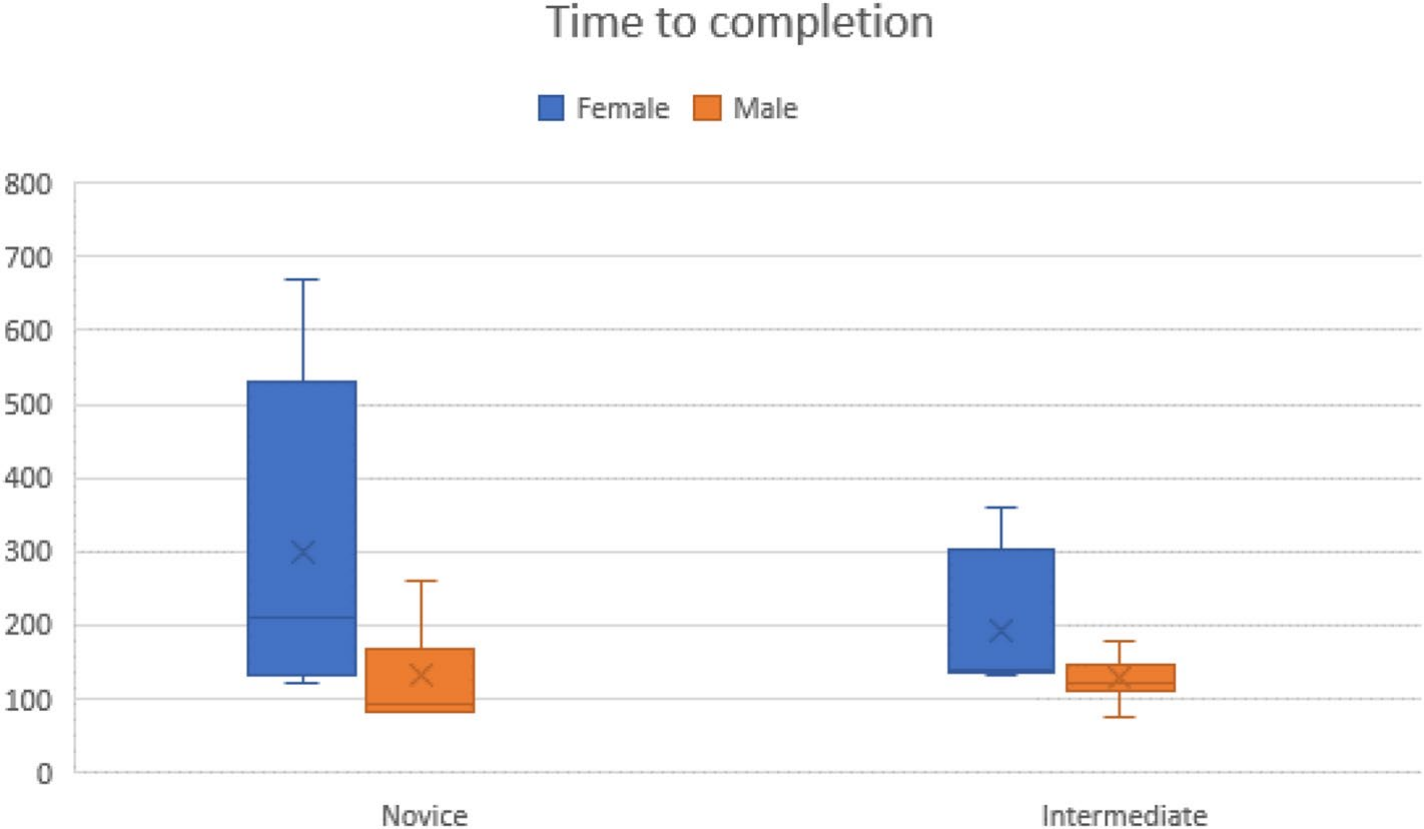
Time taken to complete between genders. Males took significantly less time than females across both novice and intermediate groups

There were no females in the experienced group, reflective of current trends in the proportion of gender representation across surgical specialties at the time of writing. Performances from both genders in both intermediate and novice groups were compared for analysis. Females were found to take significantly longer to complete the exercise with the grasper in their non-dominant hand (Female 263.58 s $\pm$ 192.34 vs. Male 136.47 s $\pm$ 46.01, *p* = 0.001) (Fig. 2). Sub-analysis demonstrated this difference as significant only in the novice group, with no difference found between genders in the intermediate group.

#### Multivariate analysis

Between-subject effects using multivariate analysis were carried out to assess the impact of each component on measured outcomes. The ND camera path travelled across gender was significantly different in the female group when controlling for experience (*p* = 0.017).

Hand dominance when controlling for gender was significant for ND grasping efficiency (*p* = 0.028). Level of experience when accounting for hand dominance was found to be significant, with the experienced group demonstrating higher levels of proficiency across ND grasping efficiency scores than the intermediate group (*p* = 0.04) (Table 3.).

**Table 3:** Group results

Group	Hand dominance	Gender	Gaming history	Arthroscopic experience (multivariate analysis)
Novice	Grasper path length (*p* = 0.03)	Time (*p* = 0.002) Camera length (*p* = 0.001)	NS	D Grasping efficiency (*p* = 0.013) ND camera path length (*p* = 0.006)
Intermediate		NS	NS	NA
Experienced	NS	NA	NS	ND grasping efficiency (= 0.04)

## Discussion

The highlight of this study is the finding which demonstrates grasper efficiency in the ND hand improves with increasing arthroscopic experience. This indicates that acquisition of bimanual control skills likely occurs with increased exposure to arthroscopy during conventional orthopaedic training. Thus any future implementation of simulation-based training which has been demonstrated to improve surgical skills in Orthopaedic trainees should consider the integration of bi-manual specific tasks to decrease laterality which was found in the inexperienced cohort.

Previous studies validating arthroscopic simulators available commercially have attempted to identify epitomical surgical skills reflective of expert performance. Limited availability of valid surgical simulation-based metrics to analyse performance has led to conclusions derived from endpoints with limited generalisability including; task duration [1]; surrogate skill markers to evaluate proficiency [10], and exclusion of datapoints which were incongruent with expected outcomes occasionally demonstrated [15]. This study evaluated each metric outputted from the “remove the stars” module, with exercise duration not demonstrating any significant difference between groups in either dominant or non-dominant attempts, highlighting the limitation of this endpoint as a valid metric to differentiate between levels of experience. Grasping efficiency and camera path length, however, were demonstrated to reflect levels of experience between groups; demonstrating both the ability of the simulator to readily identify between levels of experience, and the potential use of these metrics to identify acquisition of skill through Orthopaedic training.

The impact of baseline characteristics on simulated performance has been to date unreported in arthroscopic simulation-based studies. Both gender and handedness demonstrated a significant impact on simulator derived outcomes in this study. Handedness has been addressed previously in surgical training, with research to date focused primarily on the left-handed trainee [2, 13]. Up to 15% orthopaedic surgeons identify as left hand dominant [16], however, variability in the operating field in orthopaedic surgery dictated by patient pathology highlights the onus on orthopaedic surgeons and trainees to reduce laterality regardless of hand dominance to promote positive patient outcomes [7].

Gender was similarly found to impact performance. A disproportionate level of female participants had a significantly longer time taken to completion recorded during the attempt completed in the non-dominant attempt reaching a level of significance in the novice group. This finding was no longer significant in the intermediate group. While it should be noted that the significance of these findings should be noted in the context of the disparity in gender distribution across the groups, this finding echoes previous research related to gender in laparoscopic practice which found right-handed makes performed superiorly in the simulated module compared to left-handed males and all females included in the study [3], and outlines potential considerations in the implementation of future surgical curricula. The novice group consisted primarily of medical students with a variable interest in surgery as a future career. This may have impacted performance, as interest in surgery has been found to impact simulated surgical performances with a degree of heterogeneity. with some studies indicating an intention to enter surgical careers as an advantage in simulated performance [6, 11].

It is worth noting that despite no significant difference between D and ND attempts across all parameters except for grasping efficiency between the intermediate and expert groups, the expert group reported greater levels of comfort using their dominant hand indicating a degree of laterality remains beyond what simulator software derived metrics can identify. Authors feel this highlights the potential for simulation to promote continued professional development in line with technological advances and prevent surgical skill decay in consultant orthopaedic surgeons.

This study has several limitations; the disproportionate representation of females limits the generalisability of the results; however, the study demographics are reflective of current surgical training and is representative of the study population. The novice group contained the most balanced representation in gender, with females consisting of 40% of the group, similarly reflecting current medical school trends, finding a significant difference in simulator-derived metrics at this level. The ceiling effect is a recognised limitation in simulation-based studies [14]. Laterality in the expert group may not be detectable in current simulator software yet have a clinical impact on patient outcomes, and as such should be considered a potential confounder.

## Conclusion

Arthroscopic simulation-based training is a valuable learning tool for orthopaedic training. This study demonstrated that experienced orthopaedic surgeons have a greater degree of ambidexterity than intermediate or novice groups, hypothesised by authors to be conferred through conventional orthopaedic training. Consequently, incorporation of simulation-based orthopaedic training in future training curricula require integrated bimanual-dedicated modules to mirror real-world practice, as well as facilitated learning opportunities for the development of bimanual skills in everyday practice.

## Data Availability

NA.

## References

[1] Braman JP, Sweet RM, Hananel DM, Ludewig PM, Van Heest AE (2015). Development and validation of a basic arthroscopy skills simulator. Arthroscopy.

[2] Chirag Sumithra P, Bhat SPS (2021). Tips for southpaw cardiac surgery trainees. Indian J Thorac Cardiovasc Surg.

[3] Elneel FH, Carter F, Tang B, Cuschieri A (2008). Extent of innate dexterity and ambidexterity across handedness and gender: Implications for training in laparoscopic surgery. Surg Endosc.

[4] Gainotti G (2015). The influence of handedness on hemispheric representation of tools: a survey. Brain Cogn.

[5] Jacobsen ME, Gustafsson A, Jorgensen PG, Park YS, Konge L (2021). Practicing procedural skills is more effective than basic psychomotor training in knee arthroscopy: a randomized study. Orthop J Sports Med.

[6] Kassam AF, Cortez AR, Winer LK, Kuethe JW, Athota KP, Quillin RC (2020). The impact of medical student interest in surgery on clerkship performance and career choice. Am J Surg.

[7] Kong X, Yang M, Li X, Ni M, Zhang G, Chen J (2020). Impact of surgeon handedness in manual and robot-assisted total hip arthroplasty. J Orthop Surg Res.

[8] Kong X, Yang M, Ong A, Guo R, Chen J, Wang Y (2020). A Surgeon's handedness in direct anterior approach-hip replacement. BMC Musculoskelet Disord.

[9] Liu L, Zhao F, Zha G, Zheng X, Yang G, Xu S (2020). Effect of surgeon's handedness on distribution of prosthesis during primary total knee arthroplasty. Zhongguo Xiu Fu Chong Jian Wai Ke Za Zhi.

[10] Luzzi A, Hellwinkel J, O'Connor M, Crutchfield C, Lynch TS (2021). The efficacy of arthroscopic simulation training on clinical ability: a systematic review. Arthroscopy.

[11] Mitchell PB, Ostby S, Mara KC, Cohen SL, Chou B, Green IC (2019). Career interest and psychomotor aptitude among medical students. J Surg Educ.

[12] Pennington N, Redmond A, Stewart T, Stone M (2014). The impact of surgeon handedness in total hip replacement. Ann R Coll Surg Engl.

[13] Prasad NK, Kvasnovsky C, Wise ES, Kavic SM (2018). The right way to teach left-handed residents: strategies for training by right handers. J Surg Educ.

[14] Repo JP, Rosqvist E, Lauritsalo S, Paloneva J (2019). Translatability and validation of non-technical skills scale for trauma (T-NOTECHS) for assessing simulated multi-professional trauma team resuscitations. BMC Med Educ.

[15] Rose K, Pedowitz R (2015). Fundamental arthroscopic skill differentiation with virtual reality simulation. Arthroscopy.

[16] Sabharwal S, MacKenzie JS, Sterling RS, Ficke JR, LaPorte DM (2020). Left-handedness among orthopaedic surgeons and trainees. JB JS Open Access.

[17] Shore EM, Grantcharov TP, Husslein H, Shirreff L, Dedy NJ, McDermott CD (2016). Validating a standardized laparoscopy curriculum for gynecology residents: a randomized controlled trial. Am J Obstet Gynecol.

[18] Skertich NJ, Schimpke SW, Lee T, Wiegmann AL, Pillai S, Rossini C (2021). Pediatric surgery simulation-based training for the general surgery resident. J Surg Res.

[19] Yaman O, Acaroglu E (2014). Role of surgeon handedness in transpedicular screw insertion. Acta Orthop Traumatol Turc.

